# The role of ixazomib as an augmented conditioning therapy in salvage autologous stem cell transplant (ASCT) and as a post-ASCT consolidation and maintenance strategy in patients with relapsed multiple myeloma (ACCoRd [UK-MRA Myeloma XII] trial): study protocol for a Phase III randomised controlled trial

**DOI:** 10.1186/s13063-018-2524-8

**Published:** 2018-03-07

**Authors:** Alina Striha, A. John Ashcroft, Anna Hockaday, David A. Cairns, Karen Boardman, Gwen Jacques, Cathy Williams, John A. Snowden, Mamta Garg, Jamie Cavenagh, Kwee Yong, Mark T. Drayson, Roger Owen, Mark Cook, Gordon Cook

**Affiliations:** 1 Clinical Trials Research Unit (CTRU), Leeds Institute of Clinical Trials Research, University of Leeds, Leeds, UK; 2 Department of Haematology, Pinderfields General Hospital, Wakefield, UK; 3 Centre for Clinical Haematology, University Hospitals Birmingham NHS Foundation Trust, Queen Elizabeth Hospital, Queen Elizabeth Medical Centre, Birmingham, UK; 4 Centre for Clinical Haematology, Nottingham University Hospitals (City Campus), Nottingham, UK; 5 Department of Haematology, Sheffield Teaching Hospitals NHS Foundation Trust, Sheffield, UK; 6 Leicester Royal Infirmary, Leicester, UK; 7 Department of Haematology, St Bartholomew’s Hospital, London, UK; 8 Department of Haematology, University College Hospital, London, UK; 9 Myeloma Clinical Trials Unit, University of Birmingham, Birmingham, UK; 10 HMDS, St James’s University Hospital, Leeds, UK; 11 Leeds Institute of Cancer & Pathology, University of Leeds, St James’s University Hospital, Leeds, UK

**Keywords:** Multiple myeloma, ASCT, Augmented ASCT, Randomised, Haematology, Depth of response

## Abstract

**Background:**

Multiple myeloma (MM) is a plasma cell tumour with an approximate annual incidence of 4500 in the UK. Therapeutic options for patients with MM have changed in the last decade with the arrival of proteasome inhibitors and immunomodulatory drugs. Despite these options, almost all patients will relapse post first-line autologous stem cell transplantation (ASCT). First relapse management (second-line treatment) has evolved in recent years with an expanding portfolio of novel agents, driving response rates influencing the durability of response. A second ASCT, as part of relapsed disease management (salvage ASCT), has been shown to prolong the progression-free survival and overall survival following a proteasome inhibitor-containing re-induction regimen, in the Cancer Research UK-funded National Cancer Research Institute Myeloma X (Intensive) study. It is now recommended that salvage ASCT be considered for suitable patients by the International Myeloma Working Group and the National Institute for Health and Care Excellence NG35 guidance.

**Methods/design:**

ACCoRd (Myeloma XII) is a UK-nationwide, individually randomised, multi-centre, multiple randomisation, open-label phase III trial with an initial single intervention registration phase aimed at relapsing MM patients who have received ASCT in first-line treatment. We will register 406 participants into the trial to allow 284 and 248 participants to be randomised at the first and second randomisations, respectively.

All participants will receive re-induction therapy until maximal response (four to six cycles of ixazomib, thalidomide and dexamethasone). Participants who achieve at least stable disease will be randomised (1:1) to receive either ASCT_Con_, using high-dose melphalan, or ASCT_Aug_, using high-dose melphalan with ixazomib. All participants achieving or maintaining a minimal response or better, following salvage ASCT, will undergo a second randomisation (1:1) to consolidation and maintenance or observation. Participants randomised to consolidation and maintenance will receive consolidation with two cycles of ixazomib, thalidomide and dexamethasone, and maintenance with ixazomib until disease progression.

**Discussion:**

The question of how best to maximise the durability of response to salvage ASCT warrants clinical investigation. Given the expanding scope of oral therapeutic agents, patient engagement with long-term maintenance strategies is a real opportunity. This study will provide evidence to better define post-relapse treatment in MM.

**Trial registration:**

ISRCTN, ISRCTN10038996. Registered on 15 December 2016.

**Electronic supplementary material:**

The online version of this article (10.1186/s13063-018-2524-8) contains supplementary material, which is available to authorized users.

## Background

### Multiple myeloma

Multiple myeloma (MM) is a plasma cell tumour and has an annual incidence in the UK of approximately 4500 new cases [1]. Therapeutic options for patients with MM have changed in the last decade with the arrival of potent novel agents such as proteasome inhibitors and immunomodulatory drugs (IMiDs) [2]. Despite the depth of responses (DoRs) obtained with these strategies, almost all patients will relapse post first-line autologous stem cell transplantation (ASCT). The most appropriate strategy as first relapse management (second-line treatment) has evolved in recent years with an expanding portfolio of novel agents, driving response rates influencing the durability of response (DuR). A second ASCT, as part of relapsed disease management (salvage ASCT), has been shown to prolong the progression-free survival (PFS) and overall survival (OS) following a proteasome inhibitor-containing re-induction regimen, in the Cancer Research UK-funded National Cancer Research Institute (NCRI) Myeloma X (Intensive) study [3]. Updated analysis showed significant improvement in second PFS amongst a salvage ASCT group of 67 months [52, ∞) vs. 35 months [31–43] in the weekly cyclophosphamide group. This result was backed up with a reduced hazard ratio (HR) of 0.37 and a statistically significant median difference of 15 months (67 months, 95% confidence interval, CI [55, ∞) vs. 52 months, 95% CI [42, 60]) in overall survival, in favour of salvage ASCT therapy with a reduced HR of 0.56 (0.35–0.90) [4]. The salvage ASCT activity in the UK has risen as a consequence of this trial and its findings, as evidenced by the British Society of Blood and Marrow Transplantation (BSBMT) data registry analysis [5]. It is now recommended that salvage ASCT be considered for suitable patients by the International Myeloma Working Group (IMWG) [6] and the National Institute for Health and Care Excellence (NICE) NG35 guidance [7].

### Tackling the unmet needs of existing studies

Two clear areas of unmet need arose from the Myeloma X study. Firstly, although superior to non-ASCT consolidation, both the DoR and DuR post-salvage ASCT were inferior to those reported in first-line treatment. This has been reported in phase III collaborative studies and national/international transplant registries. Hence, the question remains of how to utilise novel agents to augment this effect, aiming to achieve similar DoR and DuR to those seen in first-line treatment. Furthermore, in the Myeloma X study, for patients with evidence of molecular high-risk disease (*IGH*, *TP53* and *MYC* rearrangements), the DuR post-salvage ASCT was compromised, despite obtaining similar DoR to those for patients with standard-risk disease [5].

### Therapy for relapsed MM using ASCT

Currently, there are three trials registered on the ClinicalTrials.gov website involving ASCT in the salvage setting. One study (NCT01745588) aims to compare salvage ASCT with the IMiD pomalidomide as a maintenance schedule against a non-ASCT strategy of pomalidomide, clarithromycin and dexamethasone. The second trial (NCT01242267) aims to address the augmentation of high-dose melphalan with increasing doses of thalidomide in a phase I/II setting. The third trial (NCT00938626) aims to utilise a bi-specific antibody to CD3 and CD20 to augment T cell activation post-salvage ASCT re-infusion in a phase I/II setting, again not addressing the key issues raised above. These trials will report in the early years of recruitment of this study, and their results will be monitored closely. Each of these studies is USA-based and, hence, may have limited impact on UK practice.

### Consolidation and maintenance therapy

Novel agents including proteasome inhibitors and IMiDs are now routinely utilised as part of the induction regimen prior to ASCT, and this has resulted in substantial improvements in the DoR achieved before transplant in first-line treatment. The only data representing their impact in second-line ASCT is that published by the Myeloma X study group [5]. Given that DoR is prognostic for OS, a number of studies have been conducted (or are ongoing) to investigate the use of novel agents as consolidation and maintenance therapy after first-line ASCT [8]. To date, most clinical trials have reported an increase in PFS (and even OS) in relation to post-ASCT consolidation/maintenance. The reported PFS from Myeloma X is shorter than that generally reported in first-line treatment, though Myeloma X did not use post-ASCT consolidation or maintenance.

The question of how best to maximise the DuR to salvage ASCT warrants clinical investigation. Given the expanding scope of therapeutic agents with differing modes of delivery (intravenous (IV) vs. subcutaneous (SC) vs. oral) combined with the potential for community-based therapy delivery, patient engagement with long-term maintenance strategies becomes a real opportunity. However, an evidence basis for the efficacy and thus the incorporation of further post-ASCT therapy is needed. The second question is to establish which patients benefit and, more importantly, which patients do not benefit from salvage ASCT. There is some evidence that molecular risk stratification at relapse after a prior transplant may delineate subgroups where a second transplant offers no advantage in terms of DuR. This clearly requires a more in-depth examination [9], as, currently, there is no evidence basis to make a clinical judgment about the use of consolidation or maintenance in the salvage ASCT setting.

### Ixazomib (MLN9708)

Ixazomib (NINLARO®, Takeda Pharmaceutical Company) is a next generation, small molecule inhibitor of the 20S proteasome that is under development for the treatment of MM and other haematologic and non-haematologic diseases. Inhibition of the 20S proteasome has been validated as a therapeutic target for the treatment of malignancies using VELCADE® (bortezomib) for injection [10]. In an effort to broaden activity against a wider range of tumour types and increase activity in tumour types where VELCADE has shown activity, Takeda has developed the proteasome inhibitor MLN9708 (ixazomib), formulated for both IV and oral administration. Ixazomib is structurally different from VELCADE and refers to the biologically active, boronic acid form of the drug. The emerging safety profile indicates that ixazomib is generally well tolerated. The adverse events (AEs) are consistent with the class-based effects of proteasome inhibition and are similar to what has been previously reported with VELCADE, though the severity of some, for example peripheral neuropathy, is less. While some of these potential toxicities may be severe, they can be managed by clinical monitoring and standard medical intervention or, as needed, dose modification or discontinuation.

It is therefore scientifically appropriate to utilise a proteasome inhibitor and IMiD combination as re-induction therapy to set the platform to assess the impact of conditioning augmentation and post-transplant consolidation/maintenance strategies. In designing the trial, we sought to evaluate the impact of a novel proteasome inhibitor that will facilitate both the clinical benefit and the patient experience with regards to deliverability of the treatment schema. Ixazomib, an orally active boronate-based reversible inhibitor of the proteasome, has shown single agent activity in phase I/II studies alongside combination therapy with dexamethasone and more recently IMiDs [11–13]. More recently, the combination of ixazomib and the IMiD lenalidomide was reported by Kumar and colleagues in combination with dexamethasone in the first-line setting [14]. The TOURMALINE-MM1 study on the use of oral ixazomib, lenalidomide and dexamethasone for relapsed MM showed that at approximately 15 months follow-up the ixazomib regimen group had a statistically significant PFS advantage over the group taking the placebo-containing comparison (21.4 months vs. 9.7 months, respectively), with an HR of 0.54 and a 95% CI [0.32, 0.92] favouring ixazomib, with OS data not yet mature for analysis [15]. Thalidomide, the first-in-class IMiD, is more affordable and a justifiable choice given that the only other phase III randomised controlled trial (RCT) in the setting of first relapse post-ASCT demonstrated that bortezomib gave added benefit to the combination of thalidomide and dexamethasone [6]. In addition, a significant proportion of patients entering this proposed trial could have received lenalidomide maintenance in the first-line Cancer Research UK-funded NCRI study (Myeloma XI). Ixazomib in combination with thalidomide and dexamethasone is currently being studied in the HOVON 126MM study (http://www.hovon.nl/studies/studies-per-ziektebeeld/mm.html?action=showstudie&studie_id=103&categorie_id=3), which is currently open to recruitment amongst patients not suitable for high-dose therapy. The use of ixazomib in the maintenance setting was reported by Kumar and colleagues at the American Society of Haematology meeting in December 2014, demonstrating efficacy and tolerability in previously untreated patients (http://www.bloodjournal.org/content/124/21/82). The HOVON 126MM study also includes a maintenance phase randomisation comparing ixazomib and a placebo.

## Methods/design

### Trial aims and objectives

The Myeloma XII (ACCoRd) study aims, firstly, to seek improvement in DoR through augmenting the conditioning regimen and, secondly, to induce more durable responses in the salvage setting via a consolidation/maintenance strategy. Pre-clinical models [16, 17] and first-line early phase studies have demonstrated that a proteasome inhibitor can augment the tumouricidal effects of melphalan by inhibiting the DNA-damage repair mechanism employed by malignant plasma cells to minimise the impact of melphalan [18, 19]. Such augmentation of high-dose melphalan with a proteasome inhibitor is key to delivering the impact on DoR. The augmented ASCT dosing schedule to be used within this trial is considered a pragmatic choice which somewhat reflects other augmented ASCT regimens utilising VELCADE, with the exception that ixazomib will not be given post-graft due to bioavailability concerns such as absorption. The major aim is to attempt to extend DuR towards that seen after first-line ASCT (approximately 30 months PFS) [20]. Such strategies would use ixazomib, which is orally active and thus patient-friendly. A positive result from this trial would not only change clinical practice, but would be a deliverable treatment schema which is more patient-friendly in terms of limited hospital attendances.

This study will determine whether augmenting the conditioning and adopting a consolidation/maintenance strategy can have a significant impact on the DoR and DuR in all patients deemed suitable for salvage ASCT. The impact on patients with molecular high-risk markers will also be evaluated.

The primary objectives of this study are to determine the impact on DoR: < very good partial response (VGPR) vs. ≥ VGPR when salvage ASCT conditioning is augmented by the addition of a proteasome inhibitor, as well as the influence of a consolidation and maintenance strategy on the DuR: PFS. The secondary objectives of this study are to assess OS, time to disease progression (or on trial PFS), overall response rate to ixazomib, thalidomide, dexamethasone (ITD) re-induction, time to next treatment (TTNT), second progression-free survival (PFS2), DuR, minimal residual disease (MRD) negative rate post re-induction, post-ASCT and conversion after ITD consolidation, as well as to look at engraftment kinetics, toxicity and safety of the treatment regimens and quality of life (QoL).

### Ixazomib dose rationale

The lack of a discernible relationship between body surface area (BSA) and ixazomib clearance over a relatively wide BSA range (1.4–2.6 m^2^) indicates that total systemic exposure (area under the curve, AUC), following fixed dosing, should be independent of the individual patient’s BSA. Therefore, BSA is not expected to affect maximum plasma drug concentration (C_max_) or AUC after IV administration or oral dosing, and thus fixed dosing is appropriate for both oral and IV routes of administration. The clinical development of ixazomib has therefore transitioned from the use of BSA-based dosing to fixed dosing in all recently initiated Takeda phase I/II studies (e.g. Studies C16005 phase II, C16007, C16008 and C16009). Accordingly, the starting dose of ixazomib in the ACCoRd study is a fixed dose of 4.0 mg, on the basis of the recommended dose of 2.23 mg/m^2^ (using a mean patient BSA of 1.86 m^2^ from the 2208 patients with MM in VELCADE clinical studies for conversion to a fixed dose).

### Translational research (host and biomarker exploration)

ACCoRd protocol incorporates four planned translational research studies:

- Biomarker exploration of prognosis determination: interphase fluorescent in situ hybridisation (iFISH) vs. next generation sequencing (NGS)-based molecular analysis
- MRD detection: comparative analysis of multi-parameter flow cytometry (MFC) and NGS-based molecular analysis
- Biomarker discovery for proteasome inhibitor sensitivity
- Immune biomarker discovery: exploration of immune functional assessment, including immune senescence, in relation to patient outcomes and response to therapy.

### Trial design

The ACCoRd study is a UK-based, individually randomised, multi-centre, multi-stage, open-label phase III trial with an initial single intervention registration phase aimed at patients with relapsed MM who wish to undergo second-line treatment. A total of 406 participants will be registered into the trial to allow 284 participants to be randomised at the first randomisation (R1) and 248 participants to be randomised at the second randomisation (R2).

**Fig. 1:**
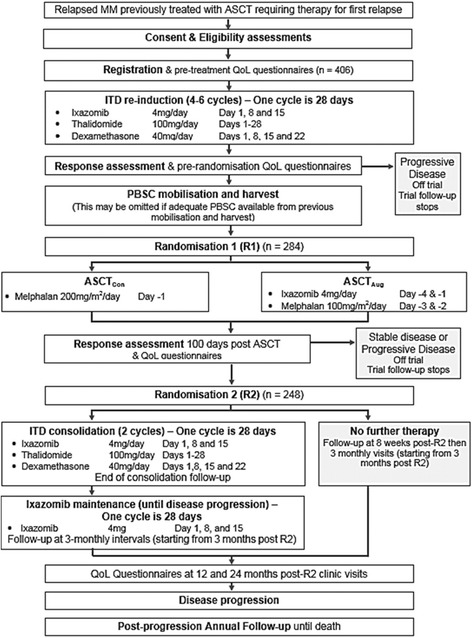
ACCoRd study flowchart

**Fig. 2:**
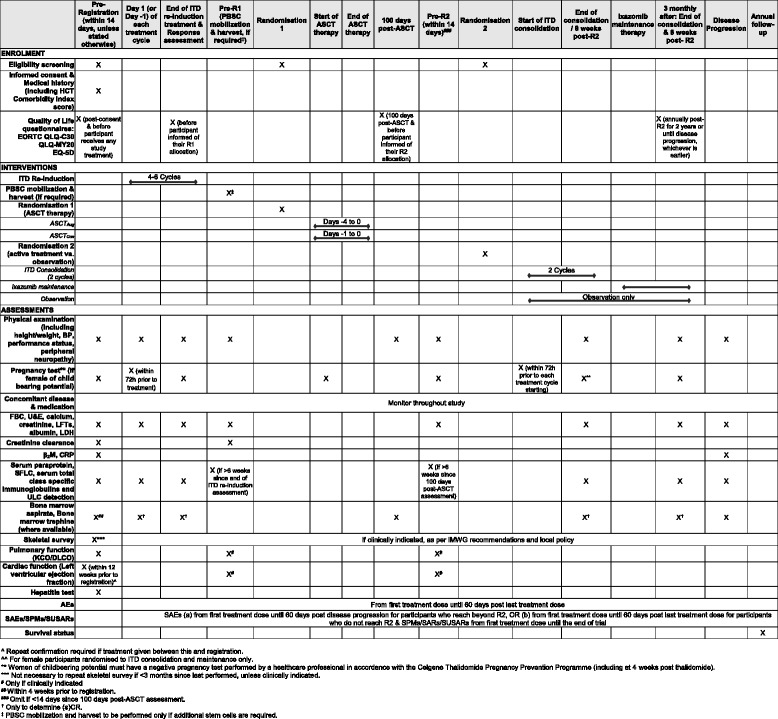
Schedule of enrolment, interventions and assessments

All participants will be registered at trial entry and will receive re-induction therapy with four to six 28-day cycles of ITD in order to reach maximum response. Participants who achieve at least stable disease (SD), as described by IMWG criteria, will be randomised on a 1:1 basis to receive either ASCT_Con_, using melphalan, or ASCT_Aug_, using melphalan along with ixazomib. All participants achieving or maintaining a minimal response (MR) or better, following trial ASCT, will undergo a second randomisation to consolidation and maintenance or no further treatment on a 1:1 basis. Participants randomised to consolidation and maintenance will receive consolidation with two 28-day cycles of ITD and maintenance with ixazomib until disease progression. Figure 1 presents a flowchart of the ACCoRd study and Figure 2 provides the schedule of enrolment, interventions and assessments for the study. Additional file 1 presents the Standard Protocol Items: Recommendations for Interventional Trials (SPIRIT) checklist.

### Trial population

The ACCoRd study aims to investigate the aforementioned treatment strategy within a population of participants with measurable, by IMWG criteria, relapsed MM requiring therapy who had undergone ASCT in first-line treatment and progressed for the first time at least 12 months after undergoing ASCT. The study will be conducted within an adult population (18 years or older), capable of giving written informed consent, with Eastern Cooperative Oncology (ECOG) Performance Status (ECOG PS) 0–2 and adequate full blood count (FBC), as well as satisfactory renal, hepatobiliary, pulmonary and cardiac function.

To be eligible for registration into the re-induction phase of the trial, participants must meet all of the inclusion criteria and none of the exclusion criteria outlined in Table 1.

**Table 1:** ACCoRd study registration inclusion and exclusion criteria

Inclusion criteria	
1. Diagnosed with relapsed MM (with measurable disease according to IMWG criteria) previously treated with ASCT) 2. First progressive disease (PD) at least 12 months following first ASCT, requiring therapy 3. Eastern Cooperative Oncology Group (ECOG) Performance Status 0–2 4. Aged at least 18 years 5. Participants must have the following blood results within 14 days before registration: (a) Absolute neutrophil count (ANC) ≥ 1 × 10^9^/L (b) Platelet count ≥ 75 × 10^9^/L. If the participant has ≥ 50% bone marrow infiltration, a platelet count of ≥ 50 × 10^9^/L is allowed. *Platelet transfusions are not allowed within 3 days before registration in order to meet these values* 6. Adequate renal function within 14 days before registration: (a) Creatinine clearance ≥ 30 ml/min (calculated according to the Cockcroft-Gault equation or other locally approved formula) 7. Adequate hepatobiliary function within 14 days before registration: (a) Total bilirubin < 2 × upper limit of normal (ULN) (b) Alanine aminotransferase (ALT) < 2 × ULN 8. Adequate pulmonary function within 14 days before registration: (a) Adequate respiratory functional reserve (delineated by carbon monoxide transfer coefficient (K_CO_)/diffusing capacity for carbon monoxide (D_LCO_) (carbon monoxide diffusion in the lung) of ≥ 50%). No evidence of a history of pulmonary disease. If a significant history, then a review by a respiratory medicine physician is required 9. Adequate cardiac function within 12 weeks before registration: (a) Left ventricular ejection fraction (LVEF) ≥ 40%. Note: repeat confirmation of cardiac function is needed if treatment is given between this assessment and registration 10. Female participants who: (a) Are not of childbearing potential, OR (b) If they are of childbearing potential, agree to practice two effective methods of contraception, at the same time, from the time of signing the informed consent form until 90 days after the last dose of study drug, OR (c) Agree to practice true abstinence when this is in line with the preferred and usual lifestyle of the subject. (Periodic abstinence [e.g. calendar, ovulation, symptothermal, post-ovulation methods] and withdrawal are not acceptable methods of contraception.) Male participants, even if surgically sterilised (i.e. status post-vasectomy), must agree to one of the following: (a) Agree to practice effective barrier contraception during the entire study treatment period and through 90 days after the last dose of study drug, OR (b) Agree to practice true abstinence when this is in line with the preferred and usual lifestyle of the subject. (Periodic abstinence [e.g. calendar, ovulation, symptothermal, post-ovulation methods] and withdrawal are not acceptable methods of contraception.) Contraception for female and male participants must be in accordance with (and with consent to) the Celgene Thalidomide Pregnancy Prevention Programme 11. If female and of childbearing potential, must have a negative pregnancy test performed by a healthcare professional in accordance with the Celgene Thalidomide Pregnancy Prevention Programme 12. Patients agree not to receive other clinical trials treatment, including investigational medicinal products (IMPs) not included in this trial, within 30 days of trial registration and throughout the duration of the trial, until disease progression 13. Able to provide written informed consent	
Exclusion criteria	
1. Received prior second-line therapy for their relapsed disease other than local radiotherapy, therapeutic plasma exchange or dexamethasone (up to a maximum of 200 mg is allowed but not within 30 days prior to registration). Radiotherapy sufficient to alleviate or control pain of local invasion is permitted, but must not be within 14 days before registration. Patients who have received hemi-body radiation or similar since relapse will not be eligible 2. ≥ Grade 2 peripheral neuropathy within 14 days before registration 3. Known HIV seropositivity 4. Known resistance, intolerance or sensitivity to any component of the planned therapies 5. Any medical or psychiatric condition which, in the opinion of the investigator, contraindicates the participant’s participation in this study 6. Previous or concurrent malignancies at other sites (excluding completely resected non-melanoma skin cancer or carcinoma in situ of any type, such as cervical cancer) 7. Pregnant, lactating or breast-feeding female participants 8. Failure to have fully recovered (i.e. ≤ Grade 1 toxicity) from the reversible effects of prior chemotherapy 9. Major surgery within 14 days before registration 10. Central nervous system involvement with myeloma 11. Ongoing or active infection requiring systemic antibiotic therapy or other serious infection within 14 days before registration 12. Evidence of current uncontrolled cardiovascular conditions, including uncontrolled hypertension, uncontrolled cardiac arrhythmias, symptomatic congestive heart failure, unstable angina or myocardial infarction within the past 6 months 13. Systemic treatment, within 14 days before the first dose of ixazomib, with strong CYP3A inducers (e.g. rifampin, rifapentine, rifabutin, carbamazepine, phenytoin, phenobarbital), or use of *Ginkgo biloba* or St. John’s wort 14. Known gastrointestinal (GI) disease or GI procedure that could interfere with the oral absorption or tolerance of ixazomib, including difficulty swallowing 15. Patients who have previously been treated with ixazomib or participated in a study with ixazomib whether treated with ixazomib or not 16. Participant has current or prior hepatitis B or C infection	

At the end of re-induction therapy, patients will be assessed for response. Patients achieving at least SD and fulfilling the eligibility criteria (Table 2) for continuation on trial will proceed to first randomisation (R1).

**Table 2:** ACCoRd study first randomisation inclusion and exclusion criteria

Inclusion criteria	
1. Registered into the ACCoRd trial and received four to six cycles of ITD re-induction chemotherapy according to the protocol 2. Responded [(s)CR, VGPR or MR] or have SD according to the IMWG Uniform Response Criteria for Multiple Myeloma 3. Adequate stem cell mobilisation defined as ≥ 2 × 10^6^ CD34+ cells/kg or ≥ 2 × 10^8^ peripheral blood mononuclear cells (PBMCs)/kg available for transplantation (including cells stored from a previous harvest) •Inclusion criteria 5{4}^a^, 6{5} and 7{6} specified in Table 1 apply to R1 inclusion criteria and hence are not included in this table 7. Adequate pulmonary function within 14 days before registration: (a) Adequate respiratory functional reserve (delineated by K_CO_/D_LCO_ (carbon monoxide diffusion in the lung) of ≥ 50%). No evidence of a history of pulmonary disease. If a significant history, then a review by a respiratory medicine physician is required. 8. Adequate cardiac function within 12 weeks before registration: (a) Left ventricular ejection fraction (LVEF) ≥ 40%. Note: repeat confirmation of cardiac function is needed if treatment is given between this assessment and registration	
Exclusion criteria	
1. Received any therapy for their relapsed disease other than local radiotherapy, therapeutic plasma exchange or Myeloma XII (ACCoRd) ITD treatment, prior to first randomisation. (Radiotherapy sufficient to alleviate or control pain of local invasion is permitted, but not within 14 days prior to randomisation. Participants who have received hemi-body radiation since relapse will not be eligible.) • Exclusion criteria 2{2}, 5{3} and 6{4} specified in Table 1 apply to R1 exclusion criteria and hence are not included in this table 5. Any contraindication to protocol treatment that would make the participant ineligible	

^a^5{4} notation is to be interpreted in the following way: Table 1 inclusion criteria 5 is equivalent to Table 2 inclusion criteria 4, etc.

At approximately 100 days post-ASCT, patients will be assessed for response. A minimum of 248 patients achieving or maintaining at least MR and fulfilling eligibility criteria (Table 3) for continuation on trial will proceed to second randomisation (R2).

**Table 3:** ACCoRd study second randomisation inclusion and exclusion criteria

Inclusion criteria	
1. Registered into the ACCoRd trial and received ASCT as per randomised treatment allocation according to the protocol • Inclusion criteria 2{2} (excluding SD response), 4{3}^a^, 5{4}, 6{5} and 8{7} specified in Table 2 apply to R2 eligibility criteria and hence are not included in this table 6. Adequate pulmonary function within 14 days before randomisation: (a) No clinical evidence of deterioration in pulmonary function since first randomisation. If there is evidence of clinical deterioration, then adequate respiratory functional reserve (delineated by K_CO_/D_LCO_ (carbon monoxide diffusion in the lung) of ≥ 50%) 8. If female and of childbearing potential, must have a negative pregnancy test within 14 days prior to randomisation, performed by a healthcare professional in accordance with the Celgene Thalidomide Pregnancy Prevention Programme	
Exclusion criteria	
1. Received any therapy for their relapsed disease other than local radiotherapy, therapeutic plasma exchange or Myeloma XII (ACCoRd) protocol treatment prior to second randomisation. (Radiotherapy sufficient to alleviate or control pain of local invasion is permitted, but not within 14 days prior to randomisation. Participants who have received hemi-body radiation since relapse will not be eligible.) • Exclusion criteria 2–5{2–5} specified in Table 2 apply to R2 eligibility and hence are not included in this table	

^a^4{3} notation is to be interpreted in the following way: Table 2 inclusion criteria 4 is equivalent to Table 3 inclusion criteria 3. In this case, the notation does not transfer back to Table 1

### Sample size

There will be 406 participants registered in the trial to ensure that 284 participants can pass through the first randomisation. This allows for 5% of participants having progressive disease (PD) after re-induction (1.4% had PD after bortezomib, doxorubicin and dexamethasone (PAD) re-induction in Myeloma X), 20% of participants mobilising insufficient cells for ASCT and 5% dropout overall throughout the trial.

In Myeloma X, 60% achieved VGPR response or better after ASCT. A total of 91 participants are required in each arm to detect an increase of 20% (from 60 to 80%) in ≥ VGPR rate in the ASCT_Aug_ arm with 80% power at the two-sided 5% significance level [21] for first randomisation (R1).

The described minimum clinically relevant difference is reasonable given what has been observed in other trials; e.g. an upgrade in complete responses (CRs) of 26% was observed by Roussel and colleagues where high-dose melphalan was augmented by IV bortezomib in a phase II study in the front-line setting (comparing the impact with that of matched historical controls receiving high-dose melphalan alone) [19]. Therefore, the estimation of a 20% increase in ≥ VGPR rate seems reasonable to hypothesise.

The second randomisation requires the greatest number of participants and hence determines the number of participants required to enter the first randomisation and the number of registered participants entering the trial. A total of 284 participants should be allocated at R1 to ensure 248 participants are available at the second randomisation (R2). Randomising 284 participants at R1 means that we have greater than 95% power to identify the clinically relevant difference of 20% (from 60 to 80%) in ≥ VGPR rate, or 172 participants being randomised to R1 in order to randomise at least 150 participants in R2 to power the trial at 80% to detect a smaller increase in rate of 16% (from 60 to 76%). This calculation accounts for 12.5% of participants suffering PD after ASCT or failing to achieve at least MR after re-induction and ASCT (7.8% had a response of ≤ SD after ASCT in Myeloma X).

A median PFS of 24 months was observed in the ASCT arm in Myeloma X amongst those participants achieving ≥ VGPR. R2 in ACCoRd is 3 months later than randomisation in Myeloma X; hence, the study is powered to detect a difference in median PFS of 9 months from R2 where that would be expected to be 21 months after R2 in those participants receiving no further consolidation and maintenance treatment and 30 months after R2 in those participants receiving further consolidation and maintenance. Therefore, 192 events are required for 80% power [22]. As a result, 248 participants (124 per arm) are required to detect a 33% reduction in hazard with 80% power at the two-sided 5% significance level (HR = 0.67) at minimum 2 years follow-up.

The increase in median PFS of 9 months is within the range (7–10 months) of improvements observed with maintenance therapy in first-line myeloma treatment [23, 24]. In addition, note that the proposed protocol treatment includes both further consolidation and maintenance, and hence the anticipated difference could reasonably be expected to be within this range even in participants with relapsed disease.

Increased follow-up also allows evaluation of protocol treatment and OS as a key secondary endpoint. The 248 participants randomised at R2 provide almost 80% power at a significance level of 0.05 to detect a 15% increase in OS at 5 years after R2 (from an estimated 59% in Myeloma X up to 74% in ACCoRd). A total of 96 events are required for 79% power at minimum 4 years follow-up for all participants.

The 284 participants who undergo R1 will receive randomised ASCT (conventional or augmented) treatment. At 100 days post-ASCT, these participants will be assessed for response to treatment. In order to proceed to the next stage of the trial, R2, participants must have achieved a minimum of MR (according to the IMWG criteria). Based on experience of Myeloma X, we anticipate that not all participants will maintain at least SD. In order for the study to retain enough power for the statistical analysis, we require a minimum of 248/284 participants to meet the criteria to proceed to R2. This constitutes an approximate dropout rate of 10%.

### Recruitment and consent

Participants will be recruited from 70 research centres from around the UK, which were identified via a feasibility assessment to determine the most appropriate centres to participate in the trial.

Potential participants will be identified by the clinical team at participating centres based on their diagnosis of relapsed MM and subsequently approached during standard clinic visits for management of their disease, as well as being provided with verbal and written details about the trial. Assenting patients will then be invited to provide informed, written consent. Consenting participants will be formally assessed for eligibility, and eligible participants can be registered.

Eligibility will be confirmed prior to registration and each randomisation, with the information being recorded in the participant’s medical records and on the relevant Case Report Forms (CRFs). Informed consent must be obtained and the participant must be registered prior to the participant undergoing procedures that are specific to the study and are beyond standard routine care at the participating site.

In order to proceed to R1, participants must have achieved at least SD (according to the IMWG criteria). It is foreseen that not all participants will achieve a minimum of SD. Hence, to enable the study to retain enough power for the statistical analysis of the research question associated with the second randomisation (R2), we require a minimum of 284/406 participants to proceed to R1. The anticipated dropout rate is based on previous experience in the Myeloma X trial. Participants who do not show at least SD will cease trial treatment and will receive off-trial treatment at the discretion of the treating clinician. The trial opened to recruitment in March 2017 and is due to close to recruitment in February 2022 or when the planned sample sizes have been reached, whichever is earlier.

### Registration and randomisation

Following confirmation of written informed consent and eligibility, an authorised member of staff at the trial research site will register participants immediately into the study. Registration will be performed centrally using the Leeds Clinical Trials Research Unit (CTRU) automated 24-h telephone and web registration and randomisation system (Gen24). Following successful registration, participants will undergo four to six cycles of ITD re-induction and will subsequently be assessed for response.

Following registration and re-induction therapy, an authorised member of staff at the trial research site will randomise participants into the study. Randomisation will be performed centrally using the Gen24 system. At the first randomisation (R1), following completion of ITD re-induction chemotherapy, eligible participants (see Table 2 for details) will be randomised on a 1:1 basis to augmented ASCT (ASCT_Aug_) or conventional ASCT (ASCT_Con_).

A Gen24-generated minimisation program that incorporates a random element will be used to ensure treatment groups are well balanced for specific participant characteristics. For R1 these are: β_2_-microglobulin concentration at first relapse (< 3.5, 3.5–< 5.5, ≥ 5.5 mg/L, unknown); length of first remission or plateau (< 18 months, 18–24 months or > 24 months); response to ITD re-induction therapy (< VGPR or ≥ VGPR); cytogenetic risk status at trial entry (Standard Risk, High-Risk or Unknown), as defined by Palumbo et al., in the presence of del(17p) and/or translocation t(4;14), and/or translocation t(14;16) [25] and maintenance treatment received in first-line setting (Yes or No).

At approximately 100 days following completion of trial ASCT and assessment for response, eligible participants (see Table 3 for details) will undergo the second 1:1 randomisation (R2) to receive consolidation and maintenance or no further treatment.

A Gen24-generated minimisation program that incorporates a random element will be used to ensure treatment groups are well balanced for the following participant characteristics for R2: allocated ASCT (conventional or augmented) and response to allocated ASCT (< VGPR or ≥ VGPR).

### Protecting against bias

The study is an open-label trial which will use an independent randomisation service, and analyses will be conducted on an intention-to-treat (ITT) basis. The trial is administered by the Chief and Principal Investigators and the CTRU in accordance with Good Clinical Practice (GCP) guidelines. Response and relapse will be assessed using the IMWG International Uniform Response Criteria using blood and urine samples (and bone marrow, where required), unless progression of myeloma occurs as an isolated bone lesion, growth of a plasmacytoma or an increase in plasma cells in the bone marrow without a change in M-protein, where a tissue histological examination will be performed.

External confirmation of disease progression and response data will be performed on samples of blood and urine at baseline, post re-induction treatment, 100 days post-transplant, post-consolidation (or 8 weeks post-R2 in the no further therapy arm) and at disease progression at a central laboratory. Samples will be forwarded by centres to relevant laboratories for central analysis. All disease progression and response data will be further confirmed by blinded clinical review coordinated by the CTRU.

### Pre-registration assessments

Assessments to be performed prior to participant registration to the ACCoRd study and starting treatment are a physical examination (including systolic and diastolic blood pressure, height, weight, BSA, vital signs, assessment for peripheral neuropathy, for example, using the Neurotoxicity Assessment Tool); ECOG PS and Revised International Staging System (R-ISS) classification; medical history (including details of concomitant disease and medication, prior chemotherapy and ASCT and previous trial participation); Haematopoietic Cell Transplantation (HCT) Comorbidity Index score; FBC; serology of hepatitis B and C; urea and electrolytes, liver function tests, albumin, lactate dehydrogenase, C-reactive protein, calcium, creatinine and β_2_-microglobulin; estimated creatinine clearance; paraprotein, serum free light chains, serum total (class-specific) immunoglobulins and urinary light chain detection (quantification where available); bone marrow aspirate and trephine (if available) within 4 weeks prior to registration; a pregnancy test; cardiac function (LVEF) within 12 weeks prior to registration; pulmonary function (*K*_CO_/*D*_LCO_) and an axial skeletal survey (performed as part of the restaging process in accordance with IMWG recommendations and local policy, and can be supplemented by computed tomography (CT), magnetic resonance imaging (MRI) or other investigation when appropriate in accordance with local practice). Additionally, for the central investigation, a set of compulsory samples must be provided to the appropriate lab, to include peripheral clotted blood (10 ml) and random urine sample (10 ml); bone marrow aspirate (2 ml) in ethylenediaminetetraacetic acid (EDTA) and bone marrow trephine (where available) in formalin, embedded in paraffin/wax block or on slides (See Fig. 2 for assessment schedule details).

For the purposes of optional translational research requiring separate consent, the following samples should be taken prior to the start of ITD, for patients who have consented to the translational research investigations: peripheral blood (20 ml) in EDTA; serum (10 ml) and bone marrow aspirate (4–5 ml).

### Intervention

All participants will be registered at trial entry and will receive re-induction therapy with four to six 28-day cycles of ixazomib (4 mg/day on days 1, 8 and 15), thalidomide (100 mg/day on days 1–28) and dexamethasone (40 mg/day on days 1, 8, 15 and 22) (ITD), in order to reach maximum response. At the end of re-induction, participants will be assessed for response and will be asked to complete the pre-randomisation QoL questionnaires, unless participants have not consented or withdrawn from the QoL study element of the trial. Participants will then go on to peripheral blood stem cell (PBSC) mobilisation and harvest, which may be omitted if adequate PBSCs are available from previous mobilisation and harvest. Following this, participants who achieve at least stable disease (SD) will be randomised on a 1:1 basis to receive either conventional ASCT (ASCT_Con_), using melphalan (200 mg/m^2^/day on day −1), or augmented ASCT (ASCT_Aug_), using melphalan with ixazomib (ixazomib 4 mg/day on days −4 and −1 and melphalan 100 mg/m^2^/day on days −3 and −2). Participants will then be assessed for response 100 days post-ASCT and asked to complete the QoL questionnaires. All participants achieving or maintaining MR or better following trial ASCT will undergo R2 to consolidation and maintenance or no further treatment. Participants randomised to R2 will receive consolidation with two 28-day cycles of ITD (ixazomib (4 mg/day on days 1, 8 and 15), thalidomide (100 mg/day on days 1–28) and dexamethasone (40 mg/day on days 1, 8, 15 and 22)) and maintenance with ixazomib (4 mg on days 1, 8, and 15) given as a 28-day cycle until disease progression. Participants will be asked to complete QoL questionnaires at 12 and 24 months post-R2 at their clinic visit, unless participants have not consented or have previously withdrawn from the QoL study (See Fig. 2 for assessment schedule details).

### Follow-up

All participants registered into the ACCoRd trial and receiving re-induction treatment will be followed up until progression, death or withdrawal from trial. Participants showing PD at the response assessment at the end of ITD re-induction will be treated off trial with trial follow-up concluded. Participants undergoing R1 and showing SD or PD at 100 days post-ASCT will be treated off trial and their disease progression and death data collected. Participants undergoing R2 with a randomisation to no further treatment will be followed up for 8 weeks post-R2 and on a 3-monthly basis thereafter until disease progression. Subsequently patients will be followed up annually until death or end of study. All R2 participants will be followed up until progression, and subsequently death on an annual basis.

### Safety reporting

Adverse events (AEs) are any untoward medical occurrences in a participant or clinical trial subject who receives a medicinal product which do not necessarily have a causal relationship with this treatment. AEs can be defined as any unintentional, unfavourable clinical signs or symptoms; any new illness or disease, or the deterioration of existing disease or illness; or any clinically relevant deterioration in any laboratory assessments or clinical tests. Due to the nature of myeloma and its treatment, participants are likely to experience several AEs throughout the course of the disease.

All AEs, both related and unrelated to myeloma treatment, occurring from the first dose of ixazomib until 60 days after treatment will be reported and will be evaluated in accordance with the National Cancer Institute Common Terminology Criteria for Adverse Events V4.03 (NCI-CTCAE).

Serious adverse events (SAEs) are defined as any untoward medical occurrences or effects that result in death; or are life threatening (at the time of the event); or require in-patient hospitalisation or prolongation of existing hospitalisation; or result in persistent or significant disability or incapacity; or result in a congenital anomaly or birth defect; or any other important medical event. For all participants who undergo R2, SAEs should be reported from the date of first dose of study drug until 60 days after the date of disease progression. For all participants who do not proceed to R2, SAEs should be reported until 60 days after the last dose of study drug. Serious adverse reactions (SARs) are to be reported from registration throughout the trial.

SARs are events that are fatal or life-threatening; require or prolong hospitalisation; are significantly or permanently disabling or incapacitating; constitute a congenital anomaly or a birth defect; or jeopardise the participant and require medical or surgical intervention to prevent one of the outcomes listed above. SARs are SAEs that are deemed to be possibly related to any dose administered of any trial treatment. Suspected unexpected serious adverse reactions (SUSARs) are SARs which are not listed in the reference safety information for that medicinal product. SARs and SUSARs should be reported from the start of treatment and for the duration of the trial. An independent Data Monitoring and Ethics Committee (DMEC) will review the safety and ethics of the trial. The CTRU will prepare detailed unblinded reports for the DMEC at approximately yearly intervals. Unblinded safety updates are also prepared for review at 6-monthly intervals whilst participants are receiving trial treatment.

### Data collection

Data will be collected using paper CRFs and entered into a validated trial database by the CTRU. A validation check program will be incorporated into the trial database to verify the data, and discrepancy reports will be generated for resolution by the investigator. Priority validations will be incorporated into the validation program to ensure that any discrepancies related to participant rights or safety are expedited to sites for resolution. Data will be monitored for quality and completeness by the CTRU. Missing data will be chased until received, and confirmed as not available, or the trial is at analysis. The CTRU/Sponsor will reserve the right to intermittently conduct source data verification exercises on a sample of participants, which will be carried out by staff from the CTRU/Sponsor. Source data verification will involve direct access to participant notes at the participating hospital sites and the central collection of copies of consent forms and other relevant investigation reports. Data will be held on a secure server at the University of Leeds and paper CRFs stored in a locked unit, both of which are accessible only to authorised trial staff.

### Statistical methods and analysis

The CTRU statisticians will be responsible for the statistical analysis, and a final statistical analysis plan will be written before any analyses are undertaken.

All analyses will be conducted on the ITT population, where participants will be included according to the treatment to which they were randomised regardless of eligibility, whether they prematurely discontinued treatment or did not comply with the regimen. A per-protocol (PP) analysis, where participants will be included according to the treatment they received, will be considered for the primary endpoints if there are a considerable number of protocol violators. The safety population will consist of all participants who receive at least one dose of the relevant study treatment.

An overall two-sided 5% significance level will be used for all efficacy endpoint comparisons. For the primary endpoints, this will be adjusted to account for the formal interim analyses. Interim statistical summaries will be presented to the DMEC in strict confidence at approximately yearly intervals.

Safety analyses will summarise all SUSARs, SARs, SAEs, AEs, ARs and treatment-related mortality rates as well as laboratory changes. Safety data will be presented by treatment group for the safety population in addition to relationship to study treatment.

Formal interim analysis for lack of benefit (or harm in the language of Freidlin and colleagues [26]) will be undertaken when 25% of required participants have completed response assessments at 100 days post-ASCT after R1 (46 patients). If the lower 95% confidence bound for the odds ratio comparing the ≥ VGPR rate in ASCT_Con_ and ASCT_Aug_ is above 1 (where an odds ratio above 1 means that the ASCT_Aug_ group is worse), then treatment in this group may be halted. Formal interim analyses for early efficacy will be undertaken when 50% of required participants have completed response assessments at 100 days post-ASCT (91 participants) after R1 and when 50% of required PFS events have been observed (96 events) in follow-up after R2. These interim analyses will be assessed against a pre-specified significance level of 0.005 indicating possible overwhelming evidence of early efficacy. All interim analysis will be advisory with changes to the study at the discretion of study oversight committees. If the interim analysis for R1 leads to changes in the study protocol, study registration would continue to answer the question posed at R2. This would be achieved by extending the registration phase to include the selected treatment option at R1 for all subsequently treated patients before R2. No other formal analysis of the study is planned before the participants have attained the primary endpoints, i.e. all randomised participants have had response evaluated 100 days post-R1 or all randomised participants have been followed up for a minimum of 2 years post-R2. The key secondary endpoint, OS, will be evaluated after all continuing participants have been followed up for a minimum of 4 years post-R2. Analysis of other endpoints will be undertaken alongside these analyses, as appropriate.

For R1, the number and proportion of participants in each response category (sCR, CR, VGPR, etc.) will be summarised by allocated treatment and exact 95% CIs will be calculated. The difference in proportions for each response category in ASCT_Con_ and ASCT_Aug_ will be presented with corresponding 95% CIs.

Allocated treatment groups will be compared with respect to the proportion achieving remission (VGPR or CR) using logistic regression to adjust for the minimisation factors. The need for PBSC re-mobilisation will be recorded and adjusted for in these analyses, as appropriate. The significance levels will be adjusted to account for the interim analyses for early efficacy. The O’Brien and Fleming α-spending function [27] will be used, suggesting α-levels of 0.047 for the final analyses and 0.005 for the interim analyses. Hence, there will be evidence to suggest superiority of ASCT_Aug_ and ASCT_Con_ if the *p* values for the initial randomisation result odds ratios are ≤ 0.047. Parameter estimates, odds ratios and corresponding 95% CIs, degrees of freedom, test statistics and *p* values will be presented for each factor in the models. Residuals and predicted values produced from the models will be examined to assess the assumptions of the statistical models.

For R2, causes of progression in all participants will be summarised, and the proportions of participants with each underlying cause will be calculated. To compare ITD consolidation and ixazomib maintenance with no treatment, Cox regression analysis will be used to analyse PFS accounting for the minimisation factors. The significance levels will be adjusted to account for the interim analyses for early efficacy. Participants who are alive and not experiencing progression will be censored at the last date known to be alive and progression-free. Parameter estimates, HRs and corresponding 95% CIs, degrees of freedom, test statistics and *p* values will be presented for each variable in the model.

The proportional hazards assumptions will be assessed by plotting the hazards over time for each treatment arm and using appropriate statistical tests. If evidence is found to support the violation of the proportional hazards assumption, then alternative appropriate analysis methods will be investigated.

Secondary endpoint analysis of OS and other time-to-event endpoints will be analysed using similar methods to those described for the PFS primary endpoint. MRD negativity will be analysed using similar methods to those described for the response primary endpoint. AEs will be summarised using CTCAE categories. QoL will be summarised using mean scores adjusted for baseline and 95% CIs for each European Organisation for Research and Treatment of Cancer (EORTC) QLQ-C30 and EORTC QLQ-MY20 module symptom, role and functioning domain at each assessment time point. Similar summaries will be produced for quality-adjusted life years (QALYs), as scored by the EQ-5D questionnaire.

A series of subgroup and exploratory analyses will be undertaken. Cytogenetic subgroups will be analysed to explore a number of specific hypotheses, including the effect on PFS, OS, time to progression and response. Some examples of what will be studied include chromosome 14 translocations and abnormalities of chromosomes 1p, 1q, 13q and 17p. In addition, other regions considered to be of interest will be analysed according to the statistical analysis plan. Other subgroup and exploratory analyses may also be performed and will be described in the statistical analysis plan or separate analyses plans related to translational work.

### Protocol amendments

The trial opened to recruitment in March 2017 using protocol version 3, dated 26 September 2016. An amendment to protocol version 4 is anticipated to be approved in March 2018, focusing on relaxing exclusion criteria 1 at registration, which stated that if the participant received dexamethasone (up to a maximum of 200 mg within 30 days prior to registration) he/she would not be eligible for the trial. Protocol version 4 will remove the 30-day restriction to allow more participants into the trial whilst maintaining the maximum dose permitted.

### Trial organisation and administration

The trial was developed by the ACCoRd Trial Management Group (TMG), with the support of the UK Myeloma Research Alliance (UK-MRA or NCRI Myeloma Subgroup). The trial is sponsored by the University of Leeds (Research & Innovation Centre/Faculty of Medicine & Health, Leeds Teaching Hospitals NHS Trust/University of Leeds, St James University Hospital, Leeds, LS9 7TF and is registered (ISRCTN10038996, EudraCT Number 2016-000905-35). A core project team, a TMG, a Trial Steering Committee (TSC) and a DMEC have been established. The independent DMEC review the safety and ethics of the trial alongside trial progress, and the overall direction is overseen by the TSC. Six-monthly interim safety reports are presented to the DMEC with a full review annually. The committee members will determine the subsequent frequency of review. The DMEC, in light of the interim data and of any advice or evidence they wish to request, will advise the TSC if there are any concerns or reasons why the trial should not continue. The results of the study will be published in peer-reviewed journals and will be presented at relevant national and international conferences. The CTRU will control the final trial dataset, and any requests for access will be reviewed by the TMG and TSC, subject to existing contractual arrangements with the funders. To maintain the scientific integrity of the trial, data will not be released prior to the end of the trial, either for trial publication or oral presentation purposes, without the permission of the TSC or the Chief Investigator.

## Discussion

The study has had an encouraging start to recruitment. The trial has been able to open centres to recruitment at a rapid pace, with 22 centres open to recruitment in the first week and 65 centres open to recruitment within 4 months. The pace of recruitment is likely due to a number of factors.

Patient attitudes towards ASCT may have changed [28], given the results of NCRI Myeloma X. Participants no longer have the option of not receiving ASCT on study. In addition, NICE guidance recommends second ASCT to eligible patients who have completed re-induction without PD and have a response duration of between 12 and 24 months after their first ASCT [7]. These factors could contribute to participants and their treating physicians being more likely to agree to participate in ACCoRd, as the control arm is the basis of national standard-of-care second-line treatment for this patient group. In addition, participants are gaining access to treatment with ixazomib, currently not available outside of clinical studies.

A wider contributing factor is potentially the successful recruitment nationally to investigator-initiated trials led by the NCRI, which have provided practice-changing results. Some of the success in recruitment could be explained by considering the excellent rate of recruitment to MRC Myeloma IX (1970 participants) and NCRI Myeloma XI (4420 participants), which have a high overlap of centres with ACCoRd. This benefits from the well-developed relationship with the coordinating CTRU that has evolved over more than 20 years.

### Trial status

The ACCoRd trial opened to recruitment in March 2017. At the time of submission, the trial had recruited 140 participants, thus surpassing predicted recruitment for February 2019, 31 participants have gone through R1 and 4 participants have gone through R2.

## Additional file

Additional file 1: The ACCoRd Trial  SPIRIT checklist. (DOCX 52 kb)

## Abbreviations

AE: Adverse event
ALT: Alanine aminotransferase
ANC: Absolute neutrophil count
AR: Adverse reaction
ASCT: Autologous stem cell transplantation
ASCT_Aug_: Autologous stem cell transplantation (augmented)
ASCT_Con_: Autologous stem cell transplantation (conventional)
AUC: Area under the curve
BSA: Body surface area
BSBMT: British Society of Blood and Marrow Transplantation
C_max_: Maximum plasma drug concentration
CR: Complete response
CRF: Case Report Form
CRUK: Cancer Research United Kingdom
CTCAE: Common Terminology Criteria for Adverse Events
CTRU: Clinical Trials Research Unit
CYP: Cytochrome P450
D_LCO_: Carbon monoxide diffusion in the lung
DMEC: Data Monitoring and Ethics Committee
DoR: Depth of response
DuR: Durability of response
ECOG: Eastern Cooperative Oncology Group
EORTC: European Organisation for Research and Treatment of Cancer
FBC: Full blood count
IMiD: Immunomodulatory drug
IMP: Investigational medicinal product
IMWG: International Myeloma Working Group
ITD: Ixazomib, thalidomide, dexamethasone
ITT: Intention-to-treat
IV: Intravenous
K_CO_: Carbon monoxide transfer coefficient
mg: Milligram
MM: Multiple myeloma
MR: Minimal response
MRD: Minimal residual disease
NCI: National Cancer Institute
NCRI: National Cancer Research Institute
NICE: National Institute for Health and Care Excellence
OS: Overall survival
PBMC: Peripheral blood mononuclear cell
PBSC: Peripheral blood stem cell
PD: Progressive disease
PFS: Progression-free survival
PFS2: Time to second progression
QoL: Quality of life
R1: Randomisation 1
R2: Randomisation 2
RCT: Randomised controlled trial
REC: Research Ethics Committee
SAE: Serious adverse event
SAR: Serious adverse reaction
SC: Subcutaneous
sCR: Stringent complete response
SD: Stable disease
SOP: Standard operating procedure
SUSAR: Suspected unexpected serious adverse reaction
TMG: Trial Management Group
TSC: Trial Steering Committee
ULN: Upper limit of normal
VGPR: Very good partial response
